# Microscale temperatures affect the incidence and implications of predator-avoidance behavior in monarch caterpillars

**DOI:** 10.1007/s00442-025-05837-7

**Published:** 2025-12-10

**Authors:** Prabhjot Singh, Louie H. Yang

**Affiliations:** Department of Entomology and Nematology, University of California, Davis, CA 95616 USA

**Keywords:** Context-dependent nonconsumptive effects, Trait mediated interactions, Predator-avoidance dropping behavior, Microclimate, Acute thermal stress

## Abstract

The nonconsumptive effects associated with the predator-avoidance behaviors of potential prey species may commonly be context-dependent. In this study, we examined how temperature differences between the phyllosphere and the ground change the consequences of predator-avoidance dropping behavior in monarch caterpillars. We hypothesized that these consequences change on both diel and seasonal timescales, and that the risk associated with exposure to potentially high ground temperatures is likely to be greater for smaller caterpillars that have less thermal inertia and movement ability. We conducted field experiments to assess the mortality risk associated with dropping to the ground for different-sized caterpillars at a wide range of ground temperatures. We also assess if a caterpillar’s probability of dropping in response to a standardized, simulated attack is consistent with adaptive expectations given variation in observed mortality risk under different conditions. We found that the survivorship consequences of dropping depend on both temperature and caterpillar size and that monarch caterpillars show context-dependent predator-avoidance behaviors consistent with changing adaptive expectations across a wide range of temperatures and body sizes. However, we also observed a potentially nonadaptive willingness to drop at high temperatures, consistent with a biological constraint imposed by increased reactivity. These results contribute to a general knowledge gap regarding the context dependence of nonconsumptive effects in nature.

**Supplementary Information:**

The online version contains supplementary material available at 10.1007/s00442-025-05837-7.

## Introduction

Predators can directly affect prey by eating them (i.e., consumptive effects) or by causing them to change aspects of their biology in response to the threat of predation (i.e., nonconsumptive effects, NCEs). Many nonconsumptive effects arise when prey species use behavioral mechanisms to avoid or escape potential predators, but these behaviors have negative effects on their survivorship, growth, or reproduction (Werner and Peacor 2003; Nelson et al. 2004; Preisser et al. 2005; Buchanan et al. 2017; Hermann and Landis 2017; Wirsing et al. 2021). It is increasingly clear that nonconsumptive effects often constitute a large proportion of the overall negative effects of predators on prey (Preisser et al. 2005; Buchanan et al. 2017), potentially including lost opportunities to feed or mate, and increases in nonpredation mortality (Roitberg and Myers 1979; Dill et al. 1990; Kim et al. 2022).

The balance of costs and benefits associated with predator-avoidance behaviors may be strongly affected by their environmental context (Lima and Dill 1990; Wirsing et al. 2021). For example, the visually dependent antipredator behaviors of Trinidadian guppies are less effective in warm, turbid waters (Zanghi et al. 2023). Similarly, lizards spend less time in predator-avoidance refuges when those refuges present less favorable temperatures (Martín 2001; Sannolo et al. 2019). Among arthropods, the adaptive value of dropping from a host plant to avoid predators may be affected by the quality of the host plant, the nature of the predation threat, and the probability of survival on the ground (Roitberg and Myers 1979; Dill et al. 1990; Losey and Denno 1998; Castellanos and Barbosa 2011; Humphreys et al. 2021).

In many of these cases, an organism’s thermal environment affects the costs and benefits of its predator-avoidance behavior, and thermal regimes are rapidly changing with global warming (Seneviratne et al. 2021). While ecologists commonly consider temperatures at meso- or macroscales, most organisms are small and experience temperature variation at microscales (Pincebourde and Woods 2020). For example, the adaptive value of predator-avoidance dropping may be mediated by the potentially large differences between ground temperatures and phyllosphere temperatures just a few centimeters away (Pincebourde and Woods 2020; Vives-Ingla et al. 2023). In the context of global warming, high ground temperatures may increase the mortality risk for insects that drop off plants to avoid predators. However, even under generally warming temperatures, cooler conditions may prevail in the morning or during cooler parts of the season. The degree to which organisms are able to adaptively modulate their predator-avoidance behaviors in response to changing thermal conditions remains unknown.

There is a need to better understand the context dependence of nonconsumptive effects under real-world conditions (Nelson et al. 2004; Hermann and Landis 2017; Wirsing et al. 2021), and field studies may be especially important to examine the effects of realistic temperature variation at small spatial and temporal scales, especially in the context of climate change (Pepi et al. 2018; Pincebourde and Woods 2020; Ma et al. 2021; Vives-Ingla et al. 2023; Yang et al. 2025). Despite early studies focused on microclimatic scales and diel temperature variation (e.g., Roitberg and Myers 1979; Losey and Denno 1998), few studies have examined the selective landscape of risk associated with microclimatic variation in the field. Of particular interest is the degree to which predator-avoidance behaviors show adaptive or nonadaptive context dependence in response to changing patterns of environmental variation.

In this study, we examine how microscale field temperatures affect the predator-avoidance dropping behavior of monarch caterpillars (*Danaus plexippus*). Monarch caterpillars often drop off their host plant in response to vibrational and tactile cues from a variety of predators, including ants, wasps, lacewings, parasitic flies, and spiders (Tautz and Markl 1978; Calvert 2004; Rayor 2004; Castellanos and Barbosa 2006, 2011; Oberhauser 2012; de Anda et al. 2015; Taylor and Yack 2019; Hermann et al. 2019; Myers et al. 2020; Lee et al. 2021; Stevenson et al. 2021; Yack and Yadav 2022). In arid environments such as our field sites in northern California (USA), ground temperatures experience strong seasonal and diel variation and commonly exceed 50 °C (Graham et al. 2012; Brandani et al. 2016; Shiflett et al. 2017; Coppernoll-Houston and Potter 2018). These high ground temperatures are a general feature of these environments, and are widely incorporated into microclimatic models of surface air temperature profiles (Kearney and Porter 2017; Buckley et al. 2023). Previous laboratory studies have observed the complete mortality of monarch caterpillars with 12-h exposures at 44 °C (Nail et al. 2015), but we are not aware of any previous studies that have examined the mortality risks associated with short exposures (e.g., on the order of minutes) to the extremely high temperatures that commonly occur on the ground. However, because monarch caterpillars increase their mass 2000-fold over development, we expected them to have greater thermal inertia and mobility when larger (Yang 2000; Kalinkat et al. 2015); as a result, we anticipated that larger caterpillars would survive high ground temperatures better than small caterpillars.

We specifically aimed to address two related questions. First, what is the mortality risk associated with dropping to the ground for different-sized caterpillars at a wide range of ground temperatures? We hypothesized that this mortality risk would increase with increasing ground temperatures, but also that the greater thermal inertia and mobility of larger caterpillars would increase their ability to relocate viable host plants before experiencing acute thermal stress. Second, is a caterpillar’s probability of dropping under different conditions consistent with adaptive expectations given their survival prospects? We hypothesized that monarch caterpillars would show predator-avoidance dropping behaviors consistent with a context-dependent adaptive strategy given observed patterns of microclimatic mortality risk (i.e., caterpillars with a higher probability of surviving on the ground would be more likely to drop).

## Methods

### Caterpillar rearing

We collected a founding population of 21 wild monarch butterfly caterpillars and 4 eggs from milkweeds at three field sites near the University of California, in Davis, California, USA (19 from the Pollinator Study Garden, 38°32′15.1′′N 121°46′22.1′′W; 5 from outdoor benches at the Orchard Park Research Greenhouse Facility, 38°32′32.7′′N 121°45′47.8′′W; and 1 from the Stebbins Cold Canyon Reserve, 38°30′30.8′′N 122°05′46.5′′W). These collections were permitted by the California Department of Fish and Wildlife under Specific Use Scientific Collection Permit S-211070001-21107-001.

From this founding population, we maintained a population of ca. 100 monarch butterfly caterpillars and adults in four 160 cm × 160 cm × 180 cm screened insect enclosures (Bugdorm-2960, MegaView Science Co., Ltd., Taichung, Taiwan). Enclosures were kept inside a thermally controlled greenhouse (mean ± SD = 25 ± 6.3 °C) at the Orchard Park Research Greenhouse Facility. Butterflies were fed red Gatorade in 145 mm-diameter petri dishes filled with 5 mm glass beads, and supplemented with flowering plants (*Asclepias fascicularis, A. curassavica, Verbena bonariensis*, and *Buddleja* sp.). Milkweed host plants (*Asclepias fascicularis* and *A. curassavica)* were provided for oviposition and larval feeding.

### Field days and site

Experimental studies were conducted on 19 field days (May 31; June 1, 2, 5, 6, 8, 13; July 5, 6, 11, 12, 13, 18, 19, 20, 25, 26, 27; August 1, 2023) in an established (3-year-old) milkweed population at the Butterfly Study Garden at UC Davis (38°32′20.2′′N 121°46′23.2′′W). This site included both showy milkweed (*Asclepias speciosa*) and narrow-leaved milkweed (*Asclepias fascicularis*) interspersed in an approximately 60-cm grid with wood mulch between plants; only the showy milkweed was used in this study due to its simpler plant architecture. This site was adjacent to an agricultural field and the university campus, and was similar in plant dispersion and thermal characteristics to other managed and unmanaged field sites in the surrounding landscape (e.g., Yang et al. 2022, 2025). Trials were conducted at a range of different times between 8 AM and 4 PM to maximize both diel and seasonal temperature variation across trials.

### Willingness-to-drop experiment

Our first experiment was conducted to determine if a caterpillar’s willingness to drop depends on its size and temperature. On each field day, 6–10 monarch caterpillars were selected to represent a wide range of available sizes from the captive population. These were transported from the greenhouse facility to the field site (approximately 5 min) in a vented white paper carton with a moist paper towel and small milkweed cuttings, and placed on one of the lower leaves of an *A. speciosa* milkweed at an approximate height of 20–30 cm. Caterpillar length was measured to the nearest 0.1 mm without contact using dial calipers, then each caterpillar was allowed to acclimate and select a microhabitat on the host plant undisturbed for 20 min. During this acclimation/habitat selection period, the temperature of the ground in shaded and unshaded areas near the plant was measured using an infrared (IR) spot thermometer (TG-56, Teledyne FLIR, Wilsonville, OR 97070, USA), and the air temperature was measured using a Type K thermocouple wire attached to the same unit. After the acclimation/habitat selection period, each caterpillar’s location on the plant (top of leaf, bottom of leaf, or stem) and sun exposure status (shaded or exposed) was recorded. The temperature of the caterpillar and the temperatures of shaded and exposed areas of the host plant were measured using the IR spot thermometer. Finally, a paintbrush was used to softly brush the legs of each caterpillar to assess its willingness to drop off the leaf in response to this standardized disturbance cue. Other studies have used similar methods to stimulate predatory attacks on caterpillars (Gentry and Dyer 2002; Cisternas et al. 2020, 2022). Each caterpillar was brushed across the legs no more than two times, and was recorded as not dropping if there was no response to the second stimuli. In some cases, caterpillars landed on another leaf or remained attached to the leaf with a silk line; these were still recorded to have dropped, even though they did not reach the ground. After this trial, all caterpillars were immediately recovered and put back into a shaded, vented white paper carton to be used in the survival experiment.

### Drop-survival experiment

Our second experiment was conducted to estimate the mortality risk of falling to the ground for different-sized caterpillars at a wide range of ground temperatures. For each survivorship trial, a caterpillar was placed on an *A. speciosa* milkweed leaf at a height of 30 cm. The caterpillar’s length was measured to the nearest 0.1 mm without contact using dial calipers, and it was given 1 min to acclimate before being nudged off the leaf with a paintbrush to force a drop. The temperature of the ground where the caterpillar landed and the distance from that point to the base of the milkweed stem was recorded. Each caterpillar (*n* = 193) was observed to assess its survival on the ground. Caterpillars that were able to move themselves to a viable host plant within 10 min were determined to have survived (*n* = 96), while caterpillars that were unable to move themselves to a viable host plant within 10 min were assessed to have died (*n* = 97). Most caterpillars that survived quickly returned to the same host plant they dropped from. The caterpillars that died rapidly became unresponsive, rigid, or changed color in ways that made the determination of mortality unambiguous.

### Statistical analysis of the willingness-to-drop and drop-survival experiments

For both experiments, we first evaluated a generalized linear model (GLM) and a generalized additive model (GAM) for each explanatory variable (caterpillar length and temperature) to determine if a nonlinear fit was supported by the data. For the willingness-to-drop experiment, we compared a binomial GAM (logit link function) with caterpillar length as a smoothed factor (three knots) and a binomial GLM (logit link function), using the caterpillar drop response as a binomial response variable. We used the simpler GLM unless the GAM had a sufficiently lower sample-size corrected Akaike Information Criterion (AICc) value (ΔAICc > 1). We then compared similar binomial GLMs and binomial GAMs using caterpillar temperature as the explanatory factor. Finally, we combined caterpillar length and caterpillar temperature into a single bivariate, binomial GAM using linear coefficients for factors that were better described by a GLM, and a three-knot smoothing function for factors that were better described by a GAM. We used an analogous approach for the drop-survival experiment, except that we used ground temperature as the relevant temperature variable, and we used survival as the binomial response variable. All of our temperature metrics (caterpillar, air, ground, and plant) were highly correlated (Electronic Supplemental Material: Figure S1). These analyses resulted in two bivariate models, a GAM describing the combined effects of caterpillar size and temperature on its willingness to drop, and a GLM describing the combined effects of caterpillar size and ground temperature on its probability of survival after landing on the ground. We used these models to predict a caterpillar’s willingness to drop and probability of survival after landing on the ground across the range of sizes and temperatures observed in this experiment.

We also considered models that included interaction terms for caterpillar size and temperature. For the willingness-to-drop experiment, this model combined a linear and smoothed term, so we modeled the interaction as a GAM with a tensor product smooth with *k* = 3 for both terms. For the drop-survival experiment, both terms were linear and were modeled in a GLM. For both experiments, AICc supported the simpler models without the interaction terms.

### Microhabitat selection experiment and analysis

Data on whether caterpillars selected shaded or sun-exposed locations after a 20-min acclimation/habitat selection period were collected as part of the willingness-to-drop experiment. To determine if this microhabitat selection was affected by caterpillar size or air temperatures, we compared binomial GLMs and binomial GAMs (logit link functions) with a binomial response variable indicating if the caterpillar chose a shaded or sun-exposed location. As before, the GLMs used a logit link function and a linear coefficient, while the GAMs used a logit link function and a smoothing function with three knots. GLMs and GAMs were compared for each explanatory factor to determine if a nonlinear fit was supported. Because neither term required a nonlinear fit, a bivariate, binomial GLM (logit link functions) was constructed to include both explanatory terms. This model was used to predict the probability that a caterpillar would be observed in a shaded or sun-exposed microhabitat location as a function of caterpillar size and the air temperature.

The weather was clear for all experimental days except one. One cloudy experimental day (June 6, 2023, 10 of 199 total observations) was excluded from the microhabitat selection analysis only, because sun-exposed microhabitats were not available. We confirmed that the sky cover was qualitatively different on this day by evaluating the estimated shortwave radiation flux density at our field site location during the experiment using the Daymet dataset (Hufkens et al. 2018; Thornton et al. 2022). This analysis confirmed that shortwave radiation flux density was below 300 W/m^2^ on this day only and was above 400 W/m^2^ on all other experimental days. These data were retained in the analysis of the willingness-to-drop and drop-survival experiments, as these experiments did not depend on a choice between shaded or sun-exposed locations.

## Results

### Willingness-to-drop experiment

**Fig. 1:**
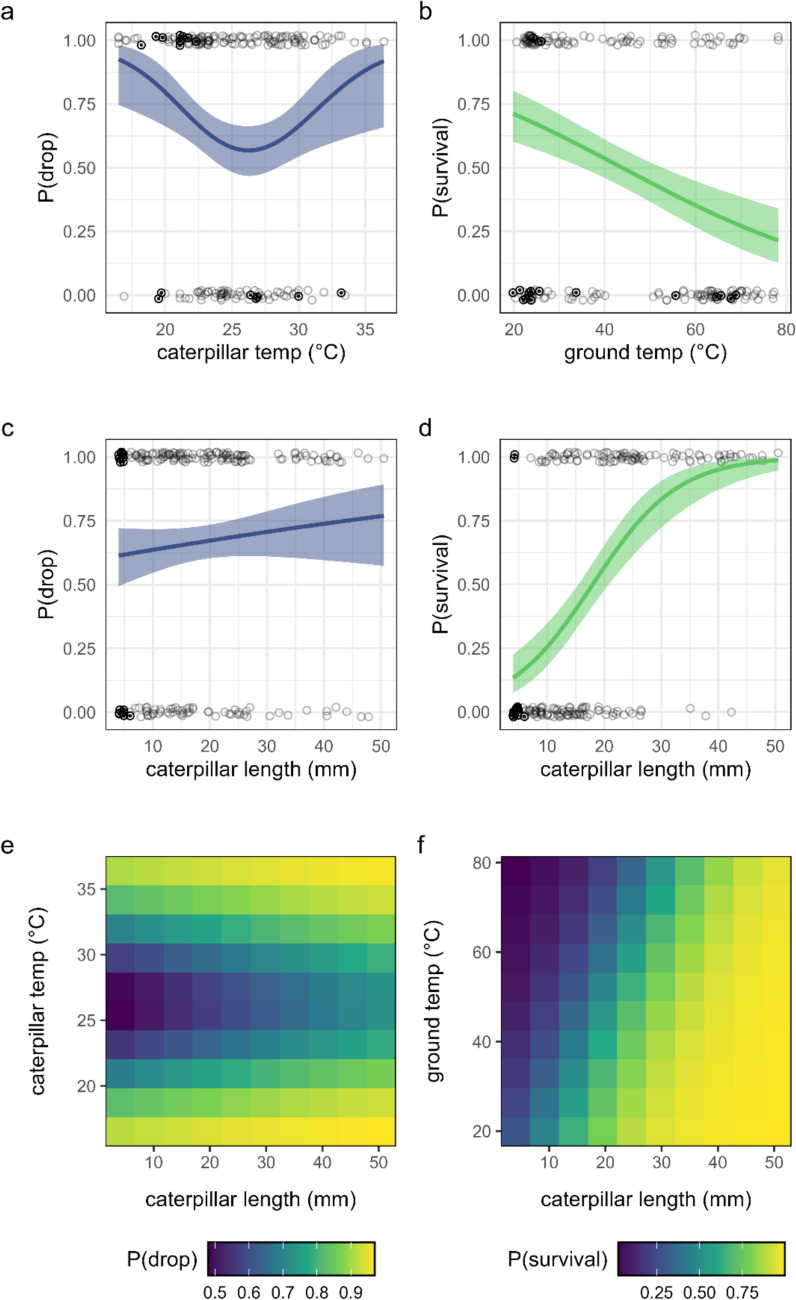
**a** Caterpillar temperature versus probability of dropping, **b** ground temperature versus probability of survival, **c** caterpillar length versus probability of dropping, **d** caterpillar length versus probability of survival, **e** predicted probability of dropping across a bivariate surface of caterpillar length and caterpillar temperature, and **f** predicted probability of drop survival across a bivariate surface of caterpillar length and ground temperature. Regression lines represent the best model fit, and the ribbon represents a 95% confidence interval. Circles represent individual caterpillars (vertically jittered for clarity). Bold circles with a center point represent first instar caterpillars

Caterpillars showed a U-shaped response to caterpillar temperature in their willingness to drop from a leaf, with a greater willingness to drop at both high and low temperatures (nonlinear, Fig. 1a). By comparison, the probability of dropping increased marginally with caterpillar size (logit-linear, Fig. 1c). In 10 of 199 observations, caterpillars dropped from their leaf but remained attached with a silk line; all were first instars. When combined in a bivariate GAM, this model predicts a sloped ridge-like surface [Fig. 1e, caterpillar temperature, *χ*^2^(1.99) = 9.2, *p* = 0.014; caterpillar length, *χ*^2^(1) = 2.6, *p* = 0.11].

### Drop-survival experiment

In this experiment, the probability of survival on the ground decreased with ground temperature (logit-linear, Fig. 1b) but increased with caterpillar length (logit-linear, Fig. 1d). In a bivariate GLM, the combined effects of these two factors predict a tilted surface with the highest probability of survival for large caterpillars at cooler temperatures, and the lowest probability of survival for small caterpillars at higher temperatures [Fig. 1f, ground temperature, *χ*^2^(1) = 22.6, *p* < 0.001; caterpillar length, *χ*^2^(1) = 63.1, *p* < 0.001].

### Microhabitat selection experiment

**Fig. 2:**
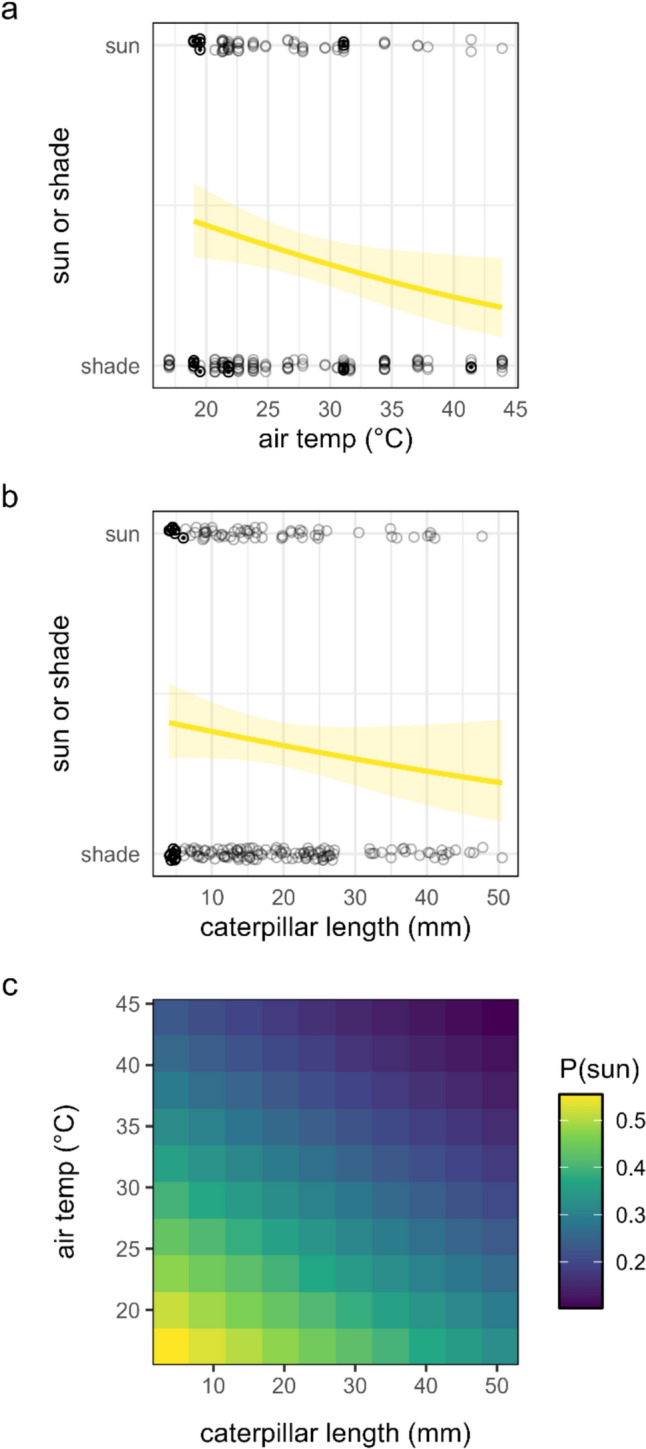
**a** Probability of sun or shade microhabitat selection as a function of air temperature and **b** caterpillar length, and **c** the predicted probability of direct sun microhabitat selection across a bivariate surface of caterpillar length and air temperature. Regression lines represent the best model fit, and the ribbon represents a 95% confidence interval. Circles represent individual caterpillars (vertically jittered for clarity). Bold circles with a center point represent first instar caterpillars

Caterpillars were more likely to be in the shade (and less likely to be in direct sun) as the air temperature increased (logit-linear, Fig. 2a). Larger caterpillars were marginally more likely to be in the shade and less likely to be in the sun than smaller caterpillars (logit-linear, Fig. 2b). A bivariate model including both factors suggests a tilted response surface with small caterpillars at cool temperatures showing the highest probability of being in the direct sunlight, and larger caterpillars at higher temperatures most likely to be in shaded microhabitats [Fig. 2c, air temperature, *χ*^2^(1) = 5.8, *p* = 0.016; caterpillar length, *χ*^2^(1) = 2.2, *p* = 0.14].

## Discussion

These results support our hypothesis that caterpillars are better able to survive dropping to the ground when ground temperatures are cooler (Fig. 1b). Caterpillars were often killed after short exposures to high ground temperatures, suggesting a high risk of mortality for dropping. Larger caterpillars were better able to survive dropping to the ground, likely due to their higher thermal inertia and greater mobility (Fig. 1d). These results are consistent with our expectations, and with our direct observations of larger caterpillars generally returning to their host plants relatively quickly, while smaller caterpillars struggled to locate a thermal refuge before experiencing visible signs of thermal stress. The combined model's predictions supported both of our key hypotheses: larger caterpillars under cooler conditions showed the highest survivorship, while smaller caterpillars under hotter conditions showed much lower survivorship (Fig. 1f).

The willingness of caterpillars to drop from a leaf seemed to increase with caterpillar size (Fig. 1c) but showed a pronounced U-shaped pattern in response to caterpillar temperatures (Fig. 1a): caterpillars were most likely to drop at very low or high temperatures, and were least likely to drop at temperatures between 25 °C and 27.5 °C. The decreasing left side of this relationship is consistent with the adaptive hypothesis that caterpillars may be more willing to drop when temperatures are lower, given the higher survivorship of caterpillars on the ground at lower temperatures (Fig. 1b). An alternative, nonadaptive hypothesis is that caterpillars at lower temperatures are less active or otherwise less capable of staying on the leaf when presented with a disturbance cue. Similarly, the increasing right side of this relationship could reflect potentially nonadaptive increases in caterpillar reactivity or decreases in coordination with increasing temperature (González-Tokman et al. 2020; Ma et al. 2021). Ectotherms generally increase their activity at higher temperatures (Halsey et al. 2015; Huey and Kingsolver 2019), and our observations in this experiment are consistent with the interpretation that caterpillars at the highest temperatures may be increasingly reactive to disturbance cues. This increase in reactivity at high temperatures seems likely to be a nonadaptive constraint given their low probability of survival on the ground under these conditions (Fig. 1a, b). This interpretation is consistent with the expected short-term “kinetic effects” of increasing temperatures on the metabolic, locomotor, and sensory processes of ectotherms (Abram et al. 2017; Yang et al. 2025), as well as the loss of neuromuscular control that can occur at above-optimal temperatures (González-Tokman et al. 2020; Ma et al. 2021). This suggests that the context-dependent, risk-sensitive adaptive behaviors of ectotherms could be fundamentally constrained by thermal physiology under extreme temperatures.

The probability of dropping increased with caterpillar size (Fig. 1c), consistent with observed patterns of survivorship on the ground (Fig. 1d). The increased willingness of larger caterpillars to drop probably reflects their higher probability of survival on the ground. Alternative (and not mutually exclusive) explanations are that this pattern could reflect size-dependent differences in predation risk or the effectiveness of dropping as a predator-avoidance behavior. For example, larger caterpillars may face a higher risk of predation due to their greater apparency, or they may drop further due to their increased weight. While the observed patterns of drop and survival probability with caterpillar size are broadly consistent, the slope of the relationship with survival (Fig. 1d) is notably steeper than the slope of the relationship with dropping probability (Fig. 1c). In particular, the probability that the smallest caterpillars will drop seems higher than expected given their extremely low probability of survival on the ground. This difference may be partly accounted for by the silk-lining behavior of first instar monarch larvae. Many caterpillars use a silk line to hang from their host plant after dropping (Sugiura and Yamazaki 2006; Castellanos and Barbosa 2006; Kim et al. 2022), and the silk-lining behavior of first instar monarch larvae reduces the probability that the smallest caterpillars will reach the ground after dropping, consistent with their low survival on the ground.

Caterpillars were more likely to select shaded microhabitats as the air temperature increased (Fig. 2a), and larger caterpillars tended to seek shaded microhabitats more than smaller caterpillars (Fig. 2b). A combined model predicted that the smallest caterpillars at the coolest temperatures had the highest probability of selecting direct sun microhabitats (Fig. 2c). One possible explanation is that smaller caterpillars are basking to accelerate their growth and development when air temperatures are cool. An alternative (but not mutually exclusive) hypothesis is that larger caterpillars may be more vulnerable to visually searching predators (e.g., birds) and are avoiding direct sun microhabitats to reduce their risk of detection (Baker 2025).

Taken together, the results of this study suggest that the survivorship consequences of dropping depend on both temperature and caterpillar size. These results contribute to a general knowledge gap regarding the context dependence of nonconsumptive effects in nature (Wirsing et al. 2021), illustrating both the potential for adaptive responses to multiple dynamic drivers and the potential for biological constraints on adaptive responses to environmental variation. These results also illustrate the potential importance of microclimatic and microhabitat variation for determining the selective landscape associated with behavioral decisions made under the risk of predation. In particular, our results emphasize that the ground and near-ground temperatures experienced by many organisms are often substantially higher than meteorological air temperatures typically measured 1.25–2 m above the ground (WMO 2023), and that microclimatic variation may be especially important in the context of heat waves and ongoing climate change. A wide range of organisms that interact with both plants and the ground may commonly grapple with the consequences of this microclimatic variation. Finally, we emphasize that this study was conducted in a field environment with realistic environmental variation on small spatial and temporal scales; similar field studies may be necessary to better understand the responses of insects and other ectotherms to the complex effects of climate change.

## Supplementary Information

Below is the link to the electronic supplementary material.

Supplementary file1 (DOCX 131 KB)

## Data Availability

The data were deposited in Dryad under the reference number http://datadryad.org/share/dONQPrrZNtV0-pvKMwv0vXzKipXaEjxvAZkdp-uCudU for peer review, forthcoming on Dryad 10.5061/dryad.1c59zw46j upon publication.

## Code availability

The code was deposited in Dryad under the reference number http://datadryad.org/share/dONQPrrZNtV0-pvKMwv0vXzKipXaEjxvAZkdp-uCudU for peer review, forthcoming on Dryad 10.5061/dryad.1c59zw46j upon publication.
