# Supplementary material for: Microscale temperatures affect the incidence and implications of predator-avoidance behavior in monarch caterpillars
Source: Oecologia. 2025 Dec 10;208(1):12. doi: 10.1007/s00442-025-05837-7 (PMC12696021; doi:10.1007/s00442-025-05837-7)
Supplement: Supplementary file 1 — Supplementary file1 (DOCX 131 KB) [file 442_2025_5837_MOESM1_ESM.docx]

**Electronic Supplemental Material**

**Figure S1.** Matrix of correlation coefficients for multiple metrics of temperature used in this study.

**
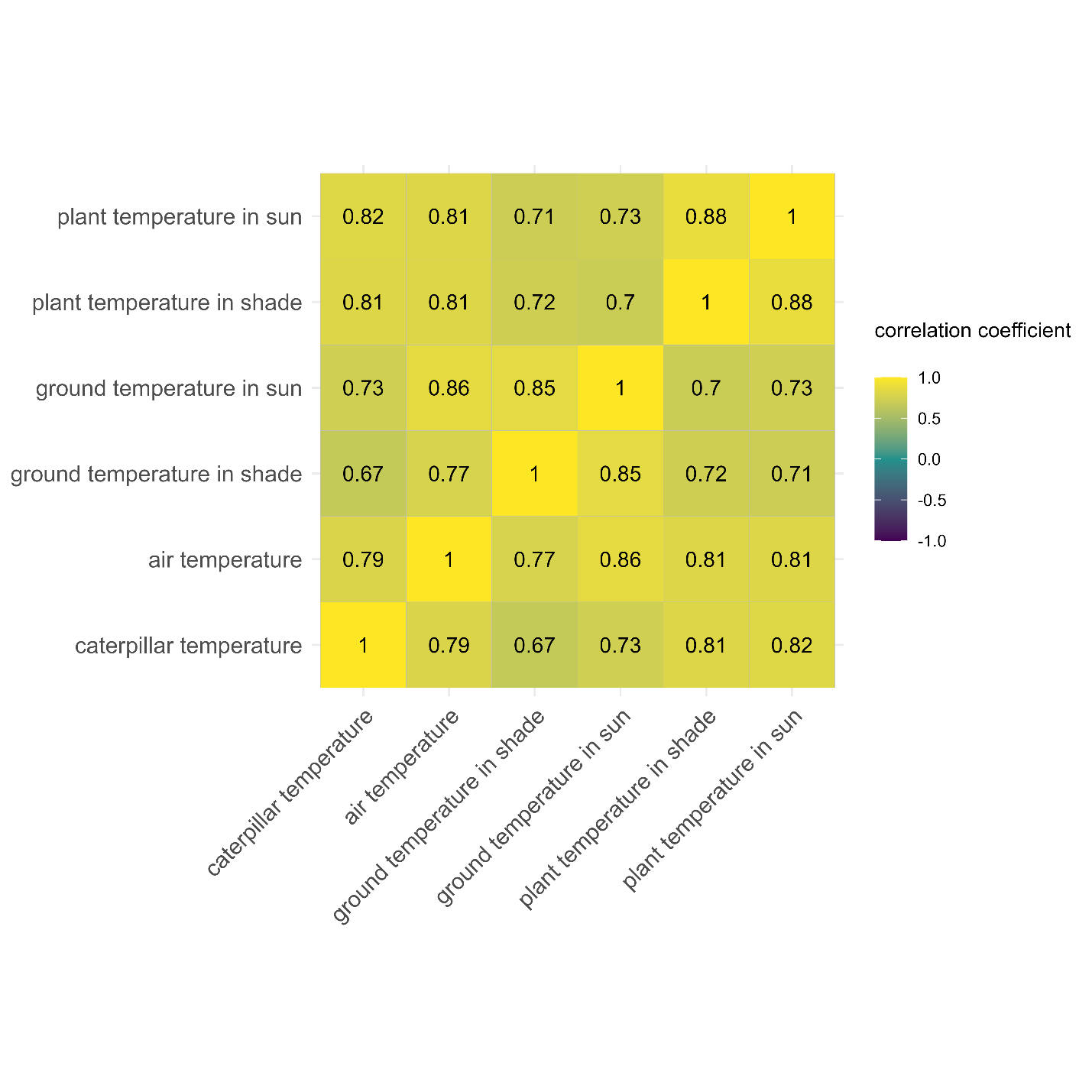
**
